# Optimization of Dye and Plasticizer Concentrations in Halochromic Sensor Films for Rapid pH Response Using Bird-Inspired Metaheuristic Algorithms

**DOI:** 10.3390/s25113494

**Published:** 2025-05-31

**Authors:** Daeuk Kim, Ronnie S. Concepcion, Joseph Rey H. Sta. Agueda, Jubert C. Marquez

**Affiliations:** 1 Department of Manufacturing Engineering and Management, Gokongwei College of Engineering, De La Salle University, Manila 1004, Philippines; ronnie.concepcion@dlsu.edu.ph (R.S.C.II); joseph.staagueda@dlsu.edu.ph (J.R.H.S.A.); 2 Center for Engineering and Sustainable Development Research, Gokongwei College of Engineering, De La Salle University, Manila 1004, Philippines; 3 Department of Biology, College of Science, De La Salle University, Manila 1004, Philippines; jubert.marquez@dlsu.edu.ph; 4 Basic Research Laboratory, Department of Physiology, College of Medicine, Smart Marine Therapeutic Center, Cardiovascular and Metabolic Disease Core Research Support Center, Inje University, Busan 47392, Republic of Korea

**Keywords:** colorimetric sensor, halochromic film, metaheuristic algorithms, optical pH sensor, optimization, smart polymer film

## Abstract

The pH level of a wound environment is a crucial biomarker for monitoring wound healing, particularly in chronic wounds, where alkalinity (pH > 7) is linked to bacterial colonization and infection. This study developed and optimized a halochromic sensor film composed of polyvinyl alcohol (PVA), polyethylene glycol (PEG), and bromothymol blue (BTB) to enable rapid and reliable pH-responsive color transitions. Feature selection using Principal Component Analysis (PCA) and the ReliefF algorithm identified Hue, Saturation, and a as key features influencing pH responsivity. Optimization of BTB (0.01–0.05%) and PEG (6–10%) concentrations was conducted using bird-inspired metaheuristic algorithms, including the Parrot Optimizer (PO), Pelican Optimization Algorithm (POA), and Secretary Bird Optimization Algorithm (SBOA). While final fitness values showed negligible variation (188.595647 for GP-PO, 188.595634 for GP-POA, and 188.595634 for GP-SBOA), GP-PO demonstrated superior convergence and stability, efficiently identifying the optimal formulation (0.02% BTB, 6% PEG). The optimized film achieved a complete color transition within 3–5 min, a 23.15% reduction compared to the non-optimized formulation. Statistical analysis revealed that BTB concentration significantly affected response time (*p* = 0.01), while PEG concentration had no significant effect (*p* > 0.05). These findings highlight the potential of halochromic films for real-time, non-invasive pH monitoring in chronic wounds.

## 1. Introduction

During wound healing, wound-tissue pH typically becomes acidic, promoting tissue regeneration. However, in chronic wounds—those characterized by prolonged healing—the pH often remains alkaline (pH > 7) due to the presence of ammonia and wound exudate, which create conditions favorable for bacterial growth [1]. The involvement of bacteria in wound pathology follows a progressive trajectory from initial contamination to colonization and, ultimately, to infection. Therefore, rising pH often serves as a sign of infections, which are considered a serious complication, as they can lead to patient mortality [2]. Studies have also reported that the pH of diabetic foot ulcers can range from approximately 6 to 9, further emphasizing the variability of wound pH in chronic conditions. Given its strong correlation with wound healing status and infection risk, pH has emerged as a crucial biomarker for the real-time monitoring and management of chronic wounds [3].

Traditional pH-measurement methods, such as glass ion-selective electrodes, are widely used for analyzing aqueous solutions. However, these conventional sensors are not suitable for monitoring wound pH due to their rigid structures and requirement for direct contact with liquid samples. Moreover, while recent developments in electronic pH sensors offer improved accuracy, the sensors often suffer from issues of invasiveness and contamination risks, making them less ideal for continuous wound assessment [4]. To address these limitations, hydrogel-based chromic sensors that undergo visible color changes in response to pH variations have been proposed as a non-invasive and biocompatible alternative for wound monitoring [5].

Chromic sensors are devices that undergo color changes in response to various stimuli. One notable advantage of these sensors is their versatility and ease of use, as they rely on simple visual color detection [6]. A prominent application of chromic sensors lies in the biomedical field, where halochromic materials—substances that change color based on pH levels—are especially important. These materials have been employed to monitor wound conditions, offering significant benefits in clinical settings. By detecting pH variations, they provide valuable information that aids medical practitioners in determining the appropriate course of treatment [7].

Natural dyes, such as anthocyanins, offer an environmentally friendly alternative; however, their practical application is hindered by several limitations. These dyes have low stability, making them highly sensitive to external conditions like moisture and illumination. Furthermore, natural dyes are characterized by low efficiency, with bioavailability typically below one percent [8]. In contrast, synthetic dyes exhibit superior sensitivity and stability, making them more suitable for applications where reliability and performance are critical. Among synthetic dyes, bromothymol blue (BTB) is particularly suitable for monitoring of chronic wounds due to its pH-sensitivity range of 5.5 to 8, which closely matches the typical pH levels observed in chronic wounds [9].

Response time plays a crucial role in developing halochromic materials for wound assessment, as swift and precise evaluation is especially important in managing chronic wounds. While it has been established that dye concentration significantly influences response time [10], the majority of previous studies have primarily focused on the optimization of color viability [11,12]. In addition to dye concentration, the plasticizer plays a pivotal role in determining response time, as it influences key polymer properties, including moisture content and swelling capacity [13]. Notably, a study by [14] demonstrated that plasticizer concentration substantially affects the color response time by modulating the arrangement of polymer backbone chains, thereby facilitating improved dye penetration and entrapment within the matrix. Therefore, optimizing these parameters is important to ensure rapid and reliable performance in wound-care applications.

In polymer design, optimization becomes increasingly challenging, particularly in systems involving multiple components and highly nonlinear interactions. Traditional optimization approaches, such as probabilistic and analytical methods, encounter significant limitations, especially when the relationships among variables are highly complex. Moreover, these methods often suffer from slow convergence rates due to requirements for extended computational time. To address these challenges, classical heuristic approaches, such as Genetic Algorithms (GA) and Particle Swarm Optimization (PSO), have been widely used. GA employs crossover and mutation mechanisms to explore the solution space, but it often struggles with fine-tuning specific parameter ranges, leading to inefficient convergence in high-dimensional problems [15]. Meanwhile, PSO is known for its rapid convergence, but it is susceptible to premature convergence and stagnation in highly nonlinear landscapes, making it less reliable in avoiding local optima [16].

Bird-inspired metaheuristic algorithms, including Cuckoo Search (CS) Algorithm (CS), Eagle Strategy (ES), Parrot Optimizer (PO), Pelican Optimization Algorithm (POA), and Secretary Bird Optimization Algorithm (SBOA), balance exploration (global search) and exploitation (local refinement) to solve optimization problems efficiently. CSA, inspired by brood parasitism, uses Lévy flights for extensive exploration but converges slowly [17]. Similarly, ES mimics eagle hunting, alternating between broad search and focused exploitation and requiring careful tuning for balance [18]. In contrast, PO, POA, and SBOA prioritize adaptive learning, precision, and aggressive search, making them more effective for dynamic and constraint-heavy problems. PO applies memory-based adaptation for evolving solutions [19]; POA ensures targeted and fast convergence by adjusting its strategy [20]; and SBOA employs aggressive yet flexible movements to refine solutions quickly [21]. While CSA and ES excel in broad search spaces, PO, POA, and SBOA are better suited for fast, adaptive, and precise optimization, making them ideal for real-time or fine-tuned applications.

A key strength of PO, POA, and SBOA lies in their ability to explore multiple solutions simultaneously, which reduces the risk of premature convergence and helps avoid local optima. These algorithms also maintain diversity in the solution space, ensuring robust performance across various optimization landscapes. Furthermore, the nature-based inspiration of their origin makes them intuitive to understand and implement, with these algorithms often requiring minimal parameter tuning. Although these algorithms have not yet been applied to optimize the chemical composition of polymers, they have been effectively utilized to determine optimal parameter values in photovoltaic models [22,23]. Given the inherently non-linear nature of photovoltaic models, this indicates that these algorithms hold significant potential for optimizing the chemical concentrations of polymers to achieve desired characteristics.

The goal and major contributions of this study include the following: (1) utilization of Principal Component Analysis (PCA) and the ReliefF algorithm for feature selection to identify key color channels influencing pH responsivity; (2) application of bird-inspired metaheuristic algorithms to optimize dye and plasticizer concentrations, enabling the development of halochromic sensor films with rapid pH response; (3) comprehensive evaluation of pH responsivity, demonstrating the efficacy of the films for continuous and real-time monitoring of chronic wound environments; and (4) contribution to the development of halochromic sensor films by bridging advanced optimization techniques and polymer science for applications in chronic wound care.

## 2. Materials and Methods

### 2.1. Materials

Polyvinyl alcohol (PVA) with a viscosity of 50–60 mPa·s and a degree of hydrolysis of 97–99%, along with polyethylene glycol (PEG) 6000, were sourced from Xilong Scientific (Guangzhou, China). Bromothymol blue (BTB) was procured from Sinopharm Chemical Reagent (Beijing, China), while glutaraldehyde (50 wt.% in H_2_O) was obtained from Sigma-Aldrich (St. Louis, MI, USA). Potassium dihydrogen phosphate was supplied by HiMedia Laboratories Pvt. Ltd. (Mumbai, India), and sodium tetraborate was acquired from Dalkem Corporation (Quezon City, Philippines).

### 2.2. Synthesis of PVA−PEG−BTB Halochromic Film

**Figure 1:**
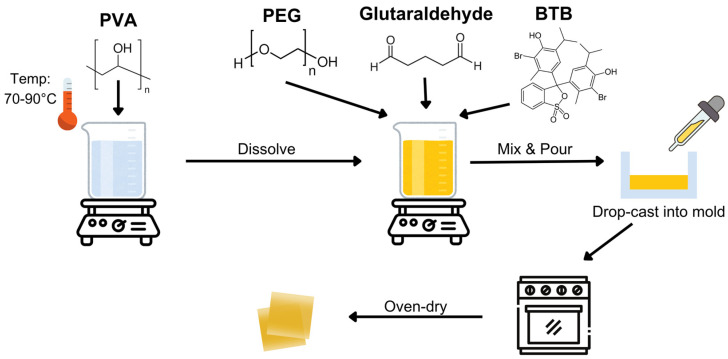
Fabrication process for halochromic sensor film employed in this study.

Figure 1 shows the process of the fabrication of the PVA−PEG−BTB film, which was based on a previously established protocol with minor modifications [24]. To prepare a 10% (*w*/*v*) PVA solution, PVA was dissolved in distilled water under a controlled temperature range of 70–90 °C. PEG solutions at varying concentrations (6%, 8%, and 10% *w*/*v*) were introduced into the mixture, and this step was followed by the addition of BTB solutions at different concentrations (1%, 3%, and 5% *w*/*v*). Finally, a fixed amount of glutaraldehyde was added to initiate crosslinking and the mixture was stirred continuously for an additional 30 min to ensure uniform crosslinking. The resulting solutions were processed using the drop-casting method and dried at 60 °C for 4 h in a Shel Lab 1600 convection oven.

**Figure 2:**
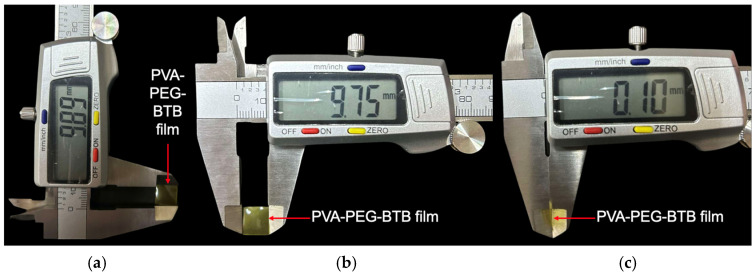
Dimensional view of the halochromic film: (**a**) length, (**b**) width, and (**c**) thickness.

A total of nine different sample combinations were formulated, representing the various pairings of PEG and BTB concentrations. For each combination, 20 sample films were fabricated, resulting in a total of 180 samples. The corresponding sample codes and concentration details are presented in Table 1. Additionally, Figure 2 presents the dimensional representation of the film, while Table 2 provides the specific measurements for each dimension.

### 2.3. Data Collection

Before data collection, buffer solutions with pH values ranging from 6 to 10 were prepared in one-unit increments to reflect the typical pH range of chronic wounds [3]. Phosphate buffers were used for pH 6 to 8, while borate buffers were utilized for pH 9 and 10. A gauze sample was immersed in each buffer solution within a Petri dish and left for 12 h before the halochromic film was tested. This step aligns with clinical recommendations for diabetic wound management, which emphasize that wound assessments should be conducted once or twice a day to monitor changes in size, depth, exudate levels, and potential signs of infection [25].

**Figure 3:**
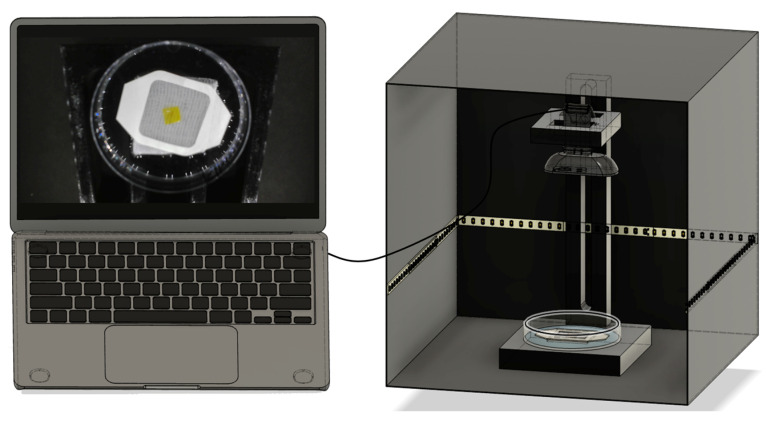
Set-up for data collection.

Following the incubation period, the film was placed inside a custom-designed data-collection platform consisting of a 3D-printed stand, a webcam, and an LED light strip enclosed within a black-colored box. The structural layout of this setup is illustrated in Figure 3. The LED strip was positioned along the sidewalls of the enclosure to minimize shadow formation and ensure uniform illumination. Image acquisition was conducted using MATLAB R2024b, which was programmed to capture video and extract one frame per second over a 10 min period. The recorded images were saved in a designated folder for further analysis.

### 2.4. Data Preparation Through Image Segmentation and Feature Selection via Principal Component Analysis (PCA) and the ReliefF Algorithm

To improve the dataset’s accuracy and dependability, graph-cut image segmentation was performed on the gathered raw images before feature selection. This method was chosen due to its ability to achieve optimal segmentation across the entire image, ensuring robust numerical performance, fast processing speed, and adaptable partitioning of weighted graphs. Graph-cut segmentation is widely utilized as an interactive segmentation algorithm; it allows precise object identification within an image by utilizing predefined foreground and background seed points.

**Figure 4:**
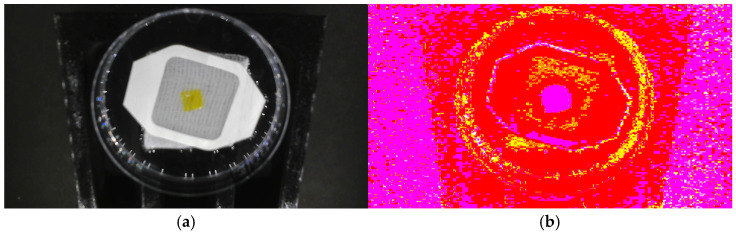

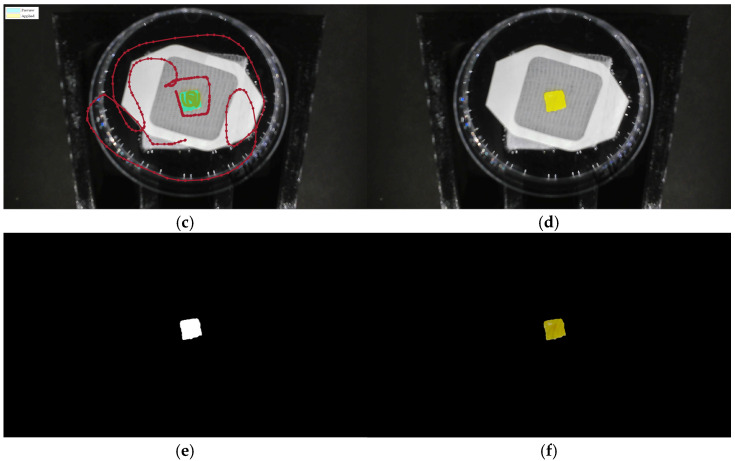
Graph-cut based image-segmentation process: (**a**) raw image, (**b**) converted Lab image, (**c**) image after lazy snapping, (**d**) selected region of interest (ROI) based on lazy snapping, (**e**) binary segmentation mask, and (**f**) final segmented image.

Figure 4 illustrates the segmentation process employed in this study, which involved multiple steps to ensure precise separation of the halochromic film from its background. First, the raw RGB images are shown in Figure 4a. These were converted to the Lab color space in Figure 4b to decouple luminance (L) from chromaticity (a and b), enhancing segmentation accuracy. To guide the segmentation process, preselected foreground and background pixel indices were used as seed points. To reduce computational complexity while preserving structural information, the image was divided into 10,468 superpixels, grouping similar pixels together. The Lab values were normalized to a [0, 1] range to ensure consistency across images before segmentation. Next, MATLAB’s lazy snapping function was applied, as shown in Figure 4c, where an interactive graph-based approach was used to mark the foreground and background regions. This process allowed for refined object-boundary selection, ensuring accurate separation of the film from its surroundings. The refined segmentation result is depicted in Figure 4d. The final step involved generating a binary segmentation mask, where pixel values of 1 represented the segmented film and 0 represented the background, as shown in Figure 4e. Finally, the segmented image was reconstructed while preserving its original colors, rather than reducing it to a black-and-white representation, as presented in Figure 4f.

From the segmented images, color values were extracted and recorded from four different color spaces—RGB, Lab, HSV, and YCbCr. These values were compiled into a cascaded dataset for feature selection. To refine the feature set, Principal Component Analysis (PCA) was conducted using Minitab software Version 22.2.0 and used to determine the number of significant features based on their contributions to variance. Subsequently, the ReliefF algorithm was utilized to assess the significance of each feature index, allocating priority weights to the most relevant features.

### 2.5. Quantitative Assessment of Color-Transition Dynamics

The data-analysis process involved reading and preprocessing time-series color data, then detecting stabilization points based on the rate of change in color parameters. The dataset contained extracted values of selected features recorded at each time point. The dataset was imported using MATLAB, ensuring that the correct variables were referenced. The relevant parameters were extracted and converted into numeric format to facilitate further computations. Any missing values were identified, and rows containing such values were removed to ensure data integrity. This process aimed to determine the time at which the color transition was completed, and this time was subsequently saved as an output variable for further analysis.

To evaluate how color parameters changed over time, the numerical gradient of each variable was calculated using the gradient function. A stabilization threshold was established wherein a slope magnitude below 0.001 was considered indicative of steady-state behavior. Furthermore, a consecutive frame criterion of 50 was applied to confirm stabilization, ensuring that transient fluctuations did not falsely indicate steady-state conditions. The stabilization points were identified using a moving-average function applied to the absolute gradient values, allowing for the detection of time frames wherein the color parameters exhibited minimal change. The detected stabilization time was recorded and stored as an output variable to be used in subsequent modeling or optimization processes.

Following the detection of stabilization points, results were analyzed and reported. If stabilization was observed, the corresponding frame number was recorded; otherwise, a notification was generated indicating the absence of stabilization within the observation period. For visual assessment of color-stabilization trends, extracted color values were plotted against time. Stabilization points were highlighted in the plots to provide a clear representation of when each parameter reached steady-state conditions.

### 2.6. Optimization and Evaluation Process

In this section, a dataset containing 180 entries with PEG concentration and BTB concentration as input variables and pH response time as the output variable was used to generate an objective function through genetic programming (GP). Genetic programming is an evolutionary algorithm inspired by natural selection, where mathematical expressions or computer programs evolve iteratively to optimize a given objective function. In this study, the objective was to minimize the pH response time, ensuring a faster color-change completion for improved sensor performance. The derived objective function that captures this relationship is presented in Equation (1). By employing genetic operators like crossover, selection, and mutation, GP continuously refined candidate solutions, evolving mathematical expressions that best captured the relationship between PEG and BTB concentrations and their influence on response time. Through this process, an optimal or near-optimal predictive expression was identified to achieve rapid color change in the halochromic sensor film.

(1) $$pH\;response\;time=f\left\{PEG\;and\;BTB\;concentrations\right\}$$

To accomplish this, specific hyperparameter settings were selected to maintain a balance between exploration and exploitation throughout the evolutionary process, as presented in Table 2. A population size of 100 was maintained across a maximum of 50 generations, ensuring a sufficient search space while preventing excessive computational cost. Tournament selection with a size of 10 was used to determine which individuals advanced in the evolutionary process, while an elite fraction of 0.1 preserved the top-performing individuals for the next generation. Additionally, a Pareto-based selection strategy was incorporated with a probability of 0.1 to maintain diversity in the evolved expressions. Structural constraints were imposed by limiting the maximum number of genes to 20 and the maximum tree depth to 10, preventing excessive model complexity. The evolutionary process was further guided by a crossover probability of 0.84, allowing offspring to inherit characteristics from parent solutions, while a mutation probability of 0.14 introduced variations to enhance exploration. These hyperparameters were carefully selected to enable genetic programming to efficiently evolve expressions that accurately predict pH response time based on PEG and BTB concentrations.

Next, the generated fitness function was utilized in three distinct bird-inspired metaheuristic optimization algorithms: the Parrot Optimizer (PO), the Pelican Optimization Algorithm (POA), and the Secretary Bird Optimization Algorithm (SBOA). The performance of each algorithm was evaluated by analyzing their convergence graphs, and the most effective algorithm was selected based on its optimization efficiency. Using the optimal PEG- and BTB-concentration values obtained from the best-performing algorithm, a new set of PVA−PEG−BTB films was fabricated and subjected to experimental validation to verify the optimization results. This methodological approach, integrating genetic programming with metaheuristic optimization, is systematically outlined in Supplementary Material S1.

### 2.7. Statistical Analysis Using ANOVA

To evaluate the effects of PEG and BTB concentrations on the pH response time of the halochromic film, a statistical analysis was conducted using a two-factor analysis of variance (ANOVA) in Microsoft Excel. ANOVA was selected to determine whether variations in PEG and BTB concentrations significantly influenced the response time and to assess any potential interaction effects between these factors. The analysis was performed with replication, as multiple pH response-time measurements were recorded for each experimental condition. A significance level (α) of 0.05 was used to assess the statistical significance of the observed differences. If the *p*-value for a factor was below this threshold, it indicated a significant influence on the response time. The results of this analysis provided statistical validation of the influence of PEG and BTB concentrations on the film’s performance.

## 3. Results and Discussion

### 3.1. Insight from Feature Selection

Principal Component Analysis (PCA) is a commonly used technique for reducing data complexity by transforming correlated variables into a set of uncorrelated components. This transformation aids in simplifying data structure, improving computational efficiency, and reducing noise while retaining most of the raw dataset’s variance. PCA is particularly advantageous in high-dimensional datasets, where redundant information may obscure key patterns and relationships [26].

**Figure 5:**
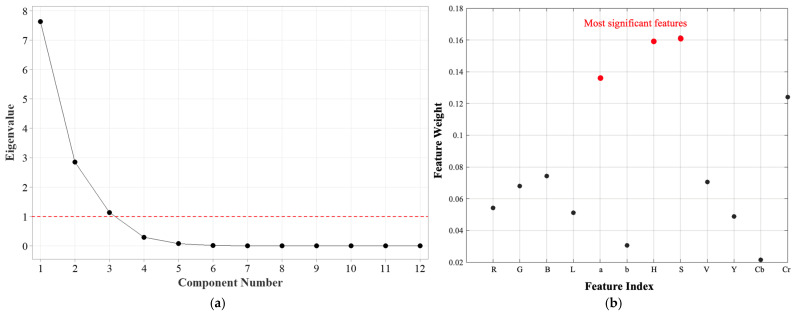
Feature-election results based on a hybrid of (**a**) principal component analysis (PCA) and (**b**) the ReliefF algorithm.

The scree plot presented in Figure 5a illustrates the eigenvalues corresponding to each principal component, providing insight into the significance of each component in explaining the variance of the dataset. The eigenvalues gradually decline, demonstrating that the initial principal components account for most of the variance, while the later components contribute only marginal additional information. A widely used approach for selecting the number of principal components to retain is the Kaiser criterion, which recommends keeping components with eigenvalues greater than 1. In the corresponding scree plot, the first three principal components exceed this threshold, indicating that they capture a significant proportion of the total variance.

The first principal component has the largest eigenvalue, signifying that it accounts for the greatest variance within the dataset. The sharp decrease in eigenvalues between the first and second components reflects a substantial drop in the amount of variance explained beyond the initial component. From the fourth component onward, the eigenvalues plateau, suggesting that these components contribute negligible additional information and may represent noise rather than meaningful variance. Based on the analysis of the scree plot, selecting three principal components is an optimal strategy for reducing dimensionality while preserving essential information. This reduction enhances computational efficiency without substantial loss of variance, making the following analyses more interpretable.

The eigenvector loadings for the first three principal components are summarized in Table 3. The results show that PC1 is primarily influenced by Red (R), Green (G), Lightness (L), and Luminance (Y), indicating that brightness and overall color intensity are key contributors to the dominant variance in pH-induced color changes. Meanwhile, PC2 is strongly associated with Blue (B) and Value (V), suggesting that these features play a crucial role in differentiating pH-induced chromatic variations. PC3 is predominantly affected by the a-channel, reinforcing the importance of red−green chromaticity in pH sensitivity.

To complement the principal component analysis (PCA), the ReliefF algorithm was employed to evaluate the relative significance of individual features within the dataset. ReliefF is a feature-selection technique that attributes weights to features according to their effectiveness in differentiating instances across distinct classes. Unlike filter-based approaches, which evaluate each feature in isolation, ReliefF accounts for the interdependencies among attributes and their role in distinguishing local instances [27]. The feature-importance plot in Figure 5b presents the weight assigned to each feature by the ReliefF algorithm. Higher weights indicate stronger relevance to the classification task, whereas lower weights suggest reduced significance.

The results highlight that the Hue (H), Saturation (S), and a channels exhibited the highest feature weights, indicating their strong discriminatory power in distinguishing different pH levels of the halochromic film. The dominance of H and S from the HSV color space suggests that the colorimetric shifts of the film are primarily dependent on hue variations and saturation intensity, aligning with the nature of the behavior of pH-sensitive dye. The findings from PCA and ReliefF are complementary in identifying the most relevant features for pH-based colorimetric sensing. PCA indicates that PC1 is mainly influenced by R, G, L, and Y, suggesting that overall color intensity and luminance variations drive the primary response to pH changes. Meanwhile, ReliefF assigns the highest weights to Hue (H), Saturation (S), and the a-channel, confirming that chromatic variations play a key role in distinguishing different pH levels. The agreement between PCA and ReliefF highlights the importance of colorimetric features over luminance-based properties in capturing pH-induced transitions in the sensor film.

The Hue channel, in particular, serves as a robust descriptor of color transformations, as it remains invariant to illumination intensity, making it ideal for consistent monitoring of color changes. The Saturation component complements the Hue channel by capturing the purity of the color shift, which is especially useful in distinguishing different pH-induced chromatic transitions. Since pH-sensitive dyes often exhibit desaturation at intermediate states, the S channel provides essential information regarding the film’s transition phase. Furthermore, the a channel from the CIELAB color space ranked among the top three features, reinforcing its relevance in describing red−green chromaticity variations.

### 3.2. Analysis of pH Response

**Figure 6:**
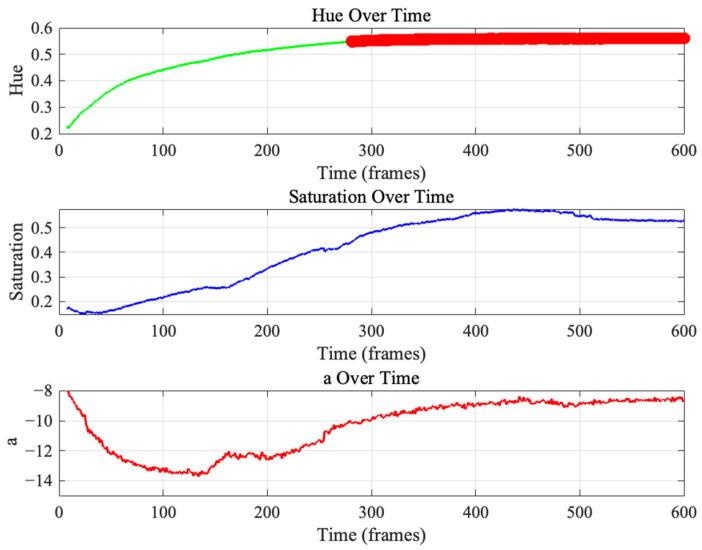
Representative stabilization curves of randomly selected halochromic film indicating completed color transition.

Figure 6 presents the stabilization curves of the randomly selected halochromic film, capturing the temporal evolution of Hue, Saturation, and a values over the course of 600 s frames. These curves illustrate the dynamic response of the film to pH changes, allowing for the assessment of the most effective color metric for detecting the transition.

Among the color parameters analyzed, Hue demonstrated the most distinct and reliable transition, exhibiting a clear sigmoidal trend with an initial increase before reaching stabilization. This pattern indicates that the Hue parameter is particularly sensitive to the film’s pH-induced color change, making it the most suitable feature for quantifying the transition. Notably, a distinct stabilization phase can be observed beyond approximately 300 frames, where the Hue curve flattens, marking the point at which the film reaches equilibrium. The latter portion of the curve, highlighted in red, signifies this stabilization phase, emphasizing the completion of the chromic response.

Similarly, the Saturation curve shows a gradual increase before reaching a plateau, though with more fluctuations compared to Hue. This suggests that while saturation changes are correlated with the chromic transition, they may be less robust in precisely determining the transition endpoint. The a parameter, derived from the CIELAB color space, exhibits a less pronounced trend, with an initial decrease followed by gradual stabilization. However, its response is less distinct compared to that observed in Hue, reinforcing the notion that Hue serves as the most effective parameter for transition detection.

Importantly, this stabilization behavior was consistently observed across all tested samples, indicating reproducibility of the chromic response and the reliability of Hue as a primary feature for monitoring the film’s pH sensitivity.

**Figure 7:**
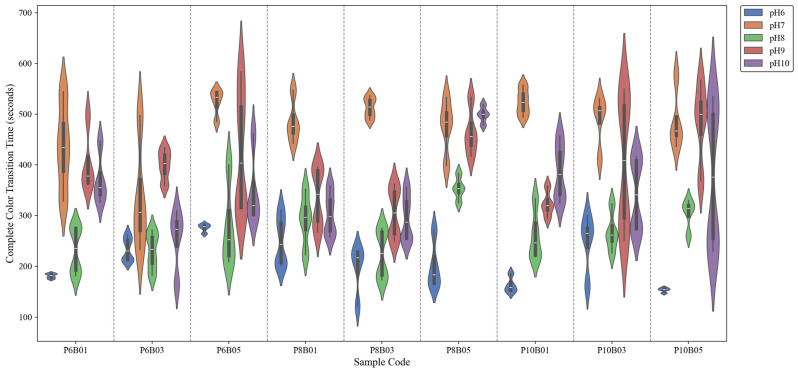
Comparison of complete color-transition times across halochromic film samples at different pH levels.

Figure 7 illustrates the distribution of complete color-transition times across various sample formulations under different pH conditions. A total of 180 samples were recorded, with 20 samples per formulation (4 per pH level across 5 pH levels) across 9 formulations. A notable trend is observed where transition time is shortest at pH 6, increases at pH 7, and then decreases again at pH 8, remaining relatively longer at pH 9 and pH 10. This pattern is consistent across all sample formulations.

The rapid transition at pH 6 can be attributed to the protonation of BTB, which shifts the dye toward its acidic form (yellow) relatively quickly. However, the increase in transition time at pH 7 suggests a buffering effect, with the system undergoing a more gradual shift between protonated and deprotonated states, leading to a slower color change. pH 7 is particularly complex compared to other pH levels, as it represents the point where proton and hydroxide ion concentrations are equal, making equilibrium shifts more gradual and causing the system to require more time to stabilize.

Among the alkaline conditions (pH 8, 9, and 10), the fastest transition is observed at pH 8. This can be explained by the optimal balance between deprotonation kinetics and dye diffusion. At pH 8, BTB transitions efficiently toward its blue form, while the polymer network remains in a hydration state that supports rapid ion exchange. As pH increases further (pH 9 and 10), the transition time slightly increases, possibly due to reduced availability of hydrogen ions and slower dye stabilization in highly alkaline conditions. The polymer environment may also contribute by altering the rate of water-mediated diffusion, which is a key factor in the chromic response.

Additionally, no distinct trend or systematic effect was observed for different concentrations of PEG and BTB, suggesting a non-linear or complex relationship between these parameters and the film’s response time. This indicates that while PEG and BTB concentrations influence the chromic behavior, their interaction with the polymer matrix and pH-dependent-diffusion processes do not follow a straightforward pattern. As a result, conventional parametric optimization approaches may be insufficient and metaheuristic algorithms may be more effective in identifying optimal formulations by navigating complex, multidimensional solution spaces.

### 3.3. Optimization Outcomes

The derived fitness function for color-completion time (pH response time), represented in Equation (2), captures the nonlinear dependencies between PEG ($x_{1}$) and BTB ($x_{2}$) concentrations and their influences on the response time. The presence of logarithmic and quadratic terms, particularly $1300\log\left(x_{1}\right)$ and $96.6{x_{1}}^{2}x_{2}$, suggests that at higher concentrations, PEG’s influence becomes more complex, potentially stabilizing or slightly increasing response time.

BTB concentration plays a significant role in modulating the response time through multiple nonlinear terms. The positive coefficient of the linear term indicates a direct increase in response time as BTB concentration rises. This observation aligns with the tendency of higher BTB levels to promote dye aggregation, which can hinder the diffusion of ions responsible for the color change. However, the function also includes complex logarithmic and quadratic dependencies, such as $-668\log\left(x_{2}\right)$ and $4x_{1}{x_{2}}^{2}$, indicating a potential threshold beyond which further increases in BTB concentration may counteract diffusion limitations and accelerate color change.

To minimize response time, an optimal balance between PEG and BTB concentrations must be achieved. The fitness function suggests that excessive BTB concentrations may slow down the response due to aggregation, whereas low or moderate PEG levels contribute to faster diffusion. The presence of logarithmic terms further suggests that there is a diminishing-returns effect at very high concentrations.

(2) $$y=4.34e+4x_{2}-123x_{1}-152\log\left(x_{1}+x_{2}+\log\left(x_{2}\right)+{x_{2}}^{2}\right)+1300\log\left(x_{1}\right)-\;668\log\left(x_{2}\right)-4.17e+4x_{1}{x_{2}}^{2}+96.6{x_{1}}^{2}x_{2}-4740$$

The optimization results, summarized in Table 4, consistently identify an optimal PEG concentration of 6% and an optimal BTB concentration of approximately 0.02%, reinforcing the reliability of the optimization framework for determining ideal formulation parameters for enhanced sensor responsiveness. While the final fitness values show negligible variation—188.595647 for GP-PO, 188.595634 for GP-POA, and 188.595634 for GP-SBOA—the faster convergence and stability of GP-PO make it the most computationally efficient approach.

**Figure 8:**
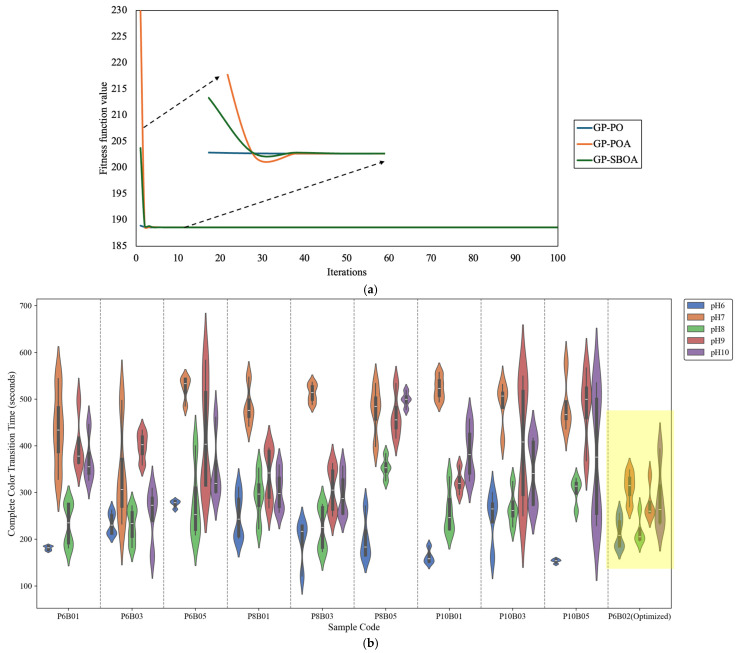
(**a**) Convergence curves of GP-PO, GP-POA, and GP-SBOA in optimizing pH response time and (**b**) comparison of complete color-transition time across halochromic film samples, including highlighted optimized results.

Figure 8a presents the convergence curves of the three optimization algorithms—GP-PO, GP-POA, and GP-SBOA—applied to minimize the color-transition time of the halochromic sensor film. All three exhibit a rapid initial decrease in the value of the fitness function, indicating efficient early-stage convergence. Among them, GP-PO stabilizes the fastest, reaching a steady state almost immediately. In contrast, GP-POA and GP-SBOA show slight oscillations before stabilizing around the third iteration, likely due to their exploration mechanisms attempting to escape local optima. Despite these fluctuations, all three algorithms ultimately converge to similar fitness values, demonstrating their robustness in solving the optimization problem.

A key aspect of this study involves the experimental evaluation of the optimized formulation (P6B02(Optimized)), highlighted in yellow in Figure 8b. The optimized film exhibits a significant reduction in transition time compared to its non-optimized counterparts. This improvement is evident across all pH levels, where the transition times are more tightly clustered and shifted toward lower values. A comparison of the average complete color-transition time between the unoptimized and optimized formulations revealed that the optimized film achieved a 23.15% faster transition. The minimization of transition time suggests enhanced film permeability and dye reactivity, which align with the primary objectives of the optimization process. The observed improvements confirm the efficacy of the employed metaheuristic optimization approach in fine-tuning dye and plasticizer concentrations to achieve rapid pH response. The reduction in transition time is especially beneficial for real-time wound-monitoring applications, where faster pH-sensitive feedback is critical for timely clinical intervention. The consistent performance across varying pH conditions further supports the robustness of the optimized formulation.

**Figure 9:**
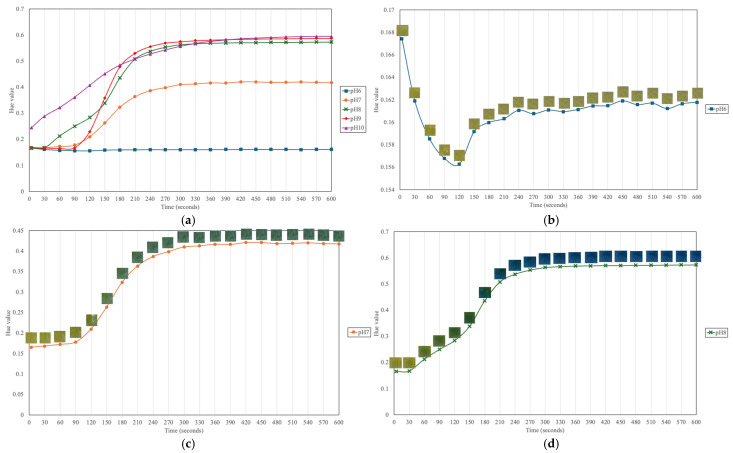

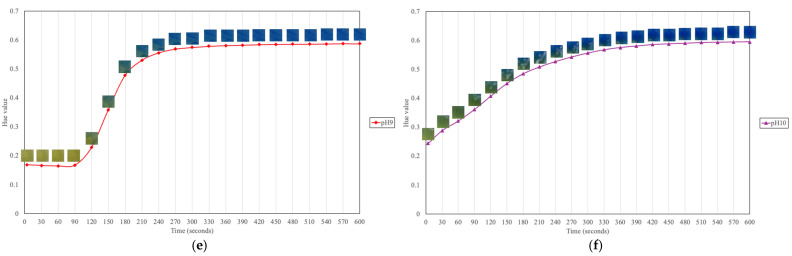
Extracted hue values of optimized PVA−PEG−BTBT film; values taken every 30 s (**a**) at all pH levels; (**b**) at pH 6; (**c**) at pH 7; (**d**) at pH 8; (**e**) at pH 9; (**f**) at pH 10.

When all plots are graphed together, as shown in Figure 9a, the hue values at pH 6 appear unchanged. However, when they are plotted separately in Figure 9b, subtle fluctuations in hue can be observed before stabilization at approximately 200 s. This suggests that while the color change at pH 6 may not be visually perceptible to the human eye, a stabilization point still exists, and this point can be detected through computer vision. Additionally, the final color states at different pH levels are distinguishable: yellow at pH 6, green at pH 7, and blue at pH 8 to 10.

As shown in Figure 9c–e, a similar color-transition pattern is observed for pH 7 to 9, where the halochromic film undergoes a rapid color change between approximately 90 and 240 s before it stabilizes, indicating the completion of the transformation. In Figure 9f, which illustrates the transition at pH 10, the film initially appears green and then gradually shifts to blue over the first half of the duration.

While the final hue values for pH 8 to 10 appear similar (blue), a measurable disparity exists: the extracted hue value is highest at pH 10, with lower values at pH 9 and then pH 8. This suggests that despite their visual similarity, these hue variations can serve as quantitative indicators for precise determination of pH. These findings highlight the film’s rapid and efficient response to alkaline environments, reinforcing its suitability for detecting pH variations in wounds, where increased alkalinity is associated with infection risk.

Table 5 presents a comparative analysis of the halochromic films from this study and previous works. While all halochromic films exhibited an initial color change within one minute of exposure to pH variations, the total time required for full color transition varied significantly across different compositions.

The halochromic films based on polylactic acid (PLA) and polyethylene glycol (PEG) that incorporated bromocresol purple and thymolphthalein as dyes exhibited times to complete color transition of 7–10 min and over 20 min, respectively. In contrast, the halochromic film developed in this study, which was composed of polyvinyl alcohol (PVA) and PEG with bromothymol blue as the pH-sensitive dye, demonstrated a significantly faster time to complete color transition of 3–5 min. The improved responsiveness of this formulation may be attributed to the hydrophilic nature of PVA, which facilitates ion diffusion, and the specific interaction of bromothymol blue with the polymer network.

This indicates that the choice of polymer matrix and dye significantly affects the response time of halochromic films. The optimization of polymer composition and dye concentration in this study significantly improved the responsiveness, resulting in a faster complete color transition.

### 3.4. Statistical Analysis

A two-factor analysis of variance (ANOVA) was conducted to assess the effects of polyethylene glycol (PEG) concentration and bromothymol blue (BTB) concentration on the response of the halochromic sensor film. The findings from the statistical analysis are presented in Table 6.

The ANOVA results suggest that PEG concentration exhibited no statistically significant influence on the response of the halochromic film, as evidenced by an F-value of 0.48, which is lower than the critical F-value of 3.05, and a corresponding *p*-value of 0.62. This suggests that variations in PEG concentration within the tested range do not contribute significantly to changes in the sensor’s response time or overall colorimetric behavior.

Conversely, the effect of BTB concentration was found to be statistically significant, with an F-value of 4.39, which exceeds the critical F-value of 3.05, and a *p*-value of 0.01. These results indicate that changes in BTB concentration significantly impact the color-change response of the sensor film, likely due to alterations in dye availability and interaction with the polymer matrix. This result highlights the significant influence of BTB concentration in enhancing the sensor’s effectiveness for pH-monitoring applications.

The interaction effect between PEG and BTB concentrations was not statistically significant, as reflected by an F-value of 0.72 (lower than the critical F-value of 2.42) and a *p*-value of 0.58. This indicates that the interaction between PEG and BTB concentrations does not have either a synergistic or antagonistic effect on the sensor’s behavior. Instead, their effects appear to be independent, with BTB concentration being the primary determinant of sensor response.

Overall, these findings suggest that BTB concentration should be carefully optimized to achieve the desired sensor performance, while variations in PEG concentration within the studied range may not substantially influence the sensor’s effectiveness. Future studies could further investigate the potential effects of PEG concentration beyond the tested range or explore alternative plasticizers to enhance the film’s response characteristics.

## 4. Conclusions

This study successfully developed and optimized a halochromic sensor film composed of polyvinyl alcohol (PVA), polyethylene glycol (PEG), and bromothymol blue (BTB) for rapid pH-responsive color transitions. By integrating feature-selection techniques (PCA, ReliefF) with advanced optimization algorithms (genetic programming and bird-inspired metaheuristic approaches), the study identified optimal dye (0.02% BTB) and plasticizer (6% PEG) concentrations, significantly enhancing the film’s pH response. Among the three optimization algorithms evaluated, the Parrot Optimizer (PO) demonstrated the best performance, exhibiting the fastest convergence and most stable optimization results. The optimized formulation achieved a complete color-transition time of 3–5 min, demonstrating a 23.15% improvement over the non-optimized formulation.

A key finding of this study is the confirmation that BTB concentration plays a critical role in accelerating response time, whereas PEG concentration within the tested range does not significantly influence the film’s performance. Furthermore, PCA and ReliefF analysis established that chromatic features (H, S, a) are more predictive of pH variations than are luminance-based properties (L, Y), reinforcing the importance of colorimetric feature selection in halochromic sensors.

Despite these advancements, the study has certain limitations. While it successfully optimizes the pH responsiveness of the sensor, long-term stability, biocompatibility, and potential interference from wound exudates remain unaddressed. Factors such as prolonged exposure to moisture, mechanical stress, and chemical interactions with wound fluids may impact sensor durability and accuracy, requiring further investigation. Although PVA and PEG are known for their biocompatibility, detailed cytotoxicity and in vivo studies are necessary to confirm the film’s suitability for direct application to wounds.

Given the significant influence of BTB concentration on response time, future research should explore the underlying mechanisms of dye−polymer interactions to further refine the film’s performance. Additionally, testing alternative plasticizers or broader concentration ranges may yield additional improvements in sensor responsiveness. Real-world validation through in vivo or clinical testing is recommended to assess the film’s efficacy in practical wound-care applications.

The integration of this halochromic sensor film with digital imaging and computer-vision-based analysis presents a promising approach for automated wound assessment and early infection detection. Overall, this study establishes a solid foundation for the further development of pH-sensitive wound-monitoring technologies, bridging polymer science, artificial-intelligence-driven optimization, and biomedical applications, and has the potential to significantly improve non-invasive wound assessment and infection detection.

## Figures and Tables

**Table 1:** Composition of halochromic sensor film with varying PEG and BTB concentrations.

Sample Code	PVA (wt/vol%)	PEG (wt/vol%)	BTB (wt/vol%)
P6B01	10	6	0.01
P6B03			0.03
P6B05			0.05
P8B01		8	0.01
P8B03			0.03
P8B05			0.05
P10B01		10	0.01
P10B03			0.03
P10B05			0.05

**Table 2:** Hyperparameter Configuration for Genetic Programming.

Hyperparameter	Value
Population size	100
Maximum generations	50
Tournament size	10
Elite fraction	0.1
Probability of pareto tournament	0.1
Maximum genes	20
Maximum tree depth	10
Crossover probability	0.84
Mutation probability	0.14

**Table 3:** Eigenvectors of the first three principal components.

Variable	PC1	PC2	PC3
R	0.355	−0.082	−0.098
G	0.335	−0.216	0.042
B	−0.074	−0.576	0.089
L	0.332	−0.231	−0.021
a	−0.183	−0.211	−0.696
b	0.314	0.291	−0.016
H	−0.324	−0.206	0.118
S	−0.226	0.0232	−0.556
V	0.223	−0.397	−0.326
Y	0.334	−0.277	−0.007
Cb	−0.314	−0.289	0.076
Cr	0.313	0.208	−0.244

**Table 4:** Results of optimization of PEG and BTB concentrations using GP-PO, GP-POA, and GP-SBOA.

Algorithm	Best Solution		Fitness Value (Minimized Color Transition Time)
	PEG Concentration	BTB Concentration	
GP-PO	6	0.020143	188.595647
GP-POA	6	0.020148	188.595634
GP-SBOA	6	0.020148	188.595634

**Table 5:** Comparison of other related studies and this work.

Base Material	Dye	Test pH Range	Time to Complete Color Transition	References
Polylactic acid (PLA), PEG	Bromocresol purple	3–11	7–10 min	[14]
Polylactic acid (PLA), PEG	Thymolphthalein	7–14	>20 min	[28]
PVA, PEG	Bromothymol blue	6–10	3–5 min	This work

**Table 6:** Two-factor ANOVA results.

Source of Variation	Sum of Squares	Degrees of Freedom	Mean Square	F	*p*-Value	F Critical
PEG concentration	13,619.34	2	6809.67	0.48	0.62	3.05
BTB concentration	123,344.60	2	61,672.30	4.39	0.01	3.05
Interaction	40,259.54	4	10,064.88	0.72	0.58	2.42
Within	2,401,405.26	171	14,043.31			
Total	2,578,628.74	179				

## Data Availability

Data are available on request to the corresponding author.

## Supplementary Materials

The following supporting information can be downloaded at: https://www.mdpi.com/article/10.3390/s25113494/s1, S1. Pseudocode of (a) Genetic Programming-Parrot Optimizer (GP-PO), (b) Genetic Programming-Pelican Optimization Algorithm (GP-POA), and (c) Genetic Programming-Secretary Bird Optimization Algorithm (SBOA).

## Abbreviations

The following abbreviations are used in this manuscript:

BTB	Bromothymol blue
PO	Parrot optimizer
POA	Pelican optimization algorithm
SBOA	Secretary bird optimization algorithm
PVA	Polyvinyl alcohol
PEG	Polyethylene glycol
PCA	Principal component analysis
GP	Genetic programming
ANOVA	Analysis of variance
PLA	Polylactic acid
