# Supplementary material for: Optimization of Dye and Plasticizer Concentrations in Halochromic Sensor Films for Rapid pH Response Using Bird-Inspired Metaheuristic Algorithms
Source: Sensors (Basel). 2025 May 31;25(11):3494. doi: 10.3390/s25113494 (PMC12158268; doi:10.3390/s25113494)
Supplement: Supplementary file 1 [file sensors-25-03494-s001.zip › sensors-3499522-supplementary.pdf]

sensors

MDPI

Article

Optimization of Dye and Plasticizer Concentrations in Halochromic Sensor Films for Rapid pH Response Using Bird-Inspired Metaheuristic Algorithms

Daeuk Kim [1,2,*], Ronnie S. Concepcion II [1,2], Joseph Rey H. Sta. Agueda [1,2] and Jubert C. Marquez [3,4]

[1] Department of Manufacturing Engineering and Management, Gokongwei College of Engineering, De La Salle University, Manila 1004, Philippines
[2] Center for Engineering and Sustainable Development Research, Gokongwei College of Engineering, De La Salle University, Manila 1004, Philippines
[3] Department of Biology, College of Science, De La Salle University, Manila 1004, Philippines
[4] Basic Research Laboratory, Department of Physiology, College of Medicine, Smart Marine Therapeutic Center, Cardiovascular and Metabolic Disease Core Research Support Center, Inje University, Busan 47392, Republic of Korea
* Correspondence: daeuk_kim@dlsu.edu.ph

Supplementary Material S1

S1. Pseudocode of (a) Genetic Programming-Parrot Optimizer (GP-PO), (b) Genetic Programming-Pelican Optimization Algorithm (GP-POA), and (c) Genetic Programming-Secretary Bird Optimization Algorithm (SBOA).

AlgorithmS1 GP-PO

```
Initialize population of symbolic expressions
Define the maximum number of generations (MaxGen) and population size (PopSize)
Define the fitness function for symbolic regression
Evaluate the fitness of each symbolic expression in the population
Identify the best symbolic expression (BestExpression) based on fitness
for generation = 1 to MaxGen do
    for each symbolic expression in the population do
        Apply genetic operations:
            - Crossover: Exchange subtrees between parent expressions
            - Mutation: Replace a random subtree with a newly generated subtree
            - Reproduction: Copy the best-performing individuals to the next generation
        Ensure offspring adhere to constraints (e.g., tree depth)
    end for
    Evaluate the fitness of the new population
    Update BestExpression if a better solution is found
    if termination criteria are met (e.g., fitness threshold or MaxGen reached) then
        break
    end if
end for
Define the best symbolic expression (BestExpression) as the ObjectiveFunction
Initialize the Parrot Optimizer parameters
Initialize population of parrots randomly within the search space
Define the maximum number of iterations (MaxIter)
Evaluate the fitness of each parrot using ObjectiveFunction
Identify the best solution (BestParrot) based on fitness
for iteration = 1 to MaxIter do
    Find the best position in the population
    for each parrot in the population do
        Select a behavior St = randint([1, 4])
        Update position based on selected behavior:
            Case St = 1: Perform Foraging Behavior
            Case St = 2: Perform Staying Behavior
            Case St = 3: Perform Communicating Behavior
            Case St = 4: Perform Fear of Strangers' Behavior
    end for
    Evaluate the fitness of the updated population using ObjectiveFunction
    Update BestParrot if a better solution is found
    if termination criteria are met (e.g., fitness threshold or MaxIter reached) then
        break
    end if
end for
return BestParrot as the optimal solution
```

(a)

AlgorithmS2 GP-POA

```
Initialize population of symbolic expressions
Define the maximum number of generations (MaxGen) and population size (PopSize)
Define the fitness function for symbolic regression
Evaluate the fitness of each symbolic expression in the population
Identify the best symbolic expression (BestExpression) based on fitness
for generation = 1 to MaxGen do
    for each symbolic expression in the population do
        Apply genetic operations:
            - Crossover: Exchange subtrees between parent expressions
            - Mutation: Replace a random subtree with a newly generated subtree
            - Reproduction: Copy the best-performing individuals to the next generation
        Ensure offspring adhere to constraints (e.g., tree depth)
    end for
    Evaluate the fitness of the new population
    Update BestExpression if a better solution is found
    if termination criteria are met (e.g., fitness threshold or MaxGen reached) then
        break
    end if
end for
Define BestExpression as the ObjectiveFunction for optimization
Initialize the Pelican Optimizer parameters
Define the POA population size (N) and the number of iterations (T)
Initialize population of pelicans randomly within the search space
Calculate the objective function for each pelican
Identify the best solution (BestPelican) based on fitness
for t = 1 to T do
    Generate prey position randomly
    for i = 1 to N do
        Moving towards prey (exploration phase)
        for j = 1 to m do
            Update the position of the j-th dimension
        end for
        Update the i-th population member
        Winging on water surface (exploitation phase)
        for j = 1 to m do
            Update the position of the j-th dimension
        end for
        Update the i-th population member
    end for
    Update best candidate solution
end for
return BestPelican as the optimal solution
```

(**b**)

AlgorithmS3 GP-SBOA

Initialize population of symbolic expressions
Define the maximum number of generations (**MaxGen**) and population size (**PopSize**)
Define the fitness function for symbolic regression
Evaluate the fitness of each symbolic expression in the population
Identify the best symbolic expression (**BestExpression**) based on fitness
for generation = 1 to MaxGen **do**
    for each symbolic expression in the population **do**
        Apply genetic operations:
            - Crossover: Exchange subtrees between parent expressions
            - Mutation: Replace a random subtree with a newly generated subtree
            - Reproduction: Copy the best-performing individuals to the next generation
        Ensure offspring adhere to constraints (e.g., tree depth)
    end for
    Evaluate the fitness of the new population
    Update **BestExpression** if a better solution is found
    if termination criteria are met (e.g., fitness threshold or MaxGen reached) **then**
        break
    end if
end for
Define **BestExpression** as the **ObjectiveFunction** for optimization
Initialize the Secretary Bird Optimization Algorithm parameters
Initialize Problem Setting $(Dim, ub, lb, Pop\_size(N)), Max\_Iter(T), Curr\_Iter(t)$
Initialize population of secretary birds randomly within the search space
Calculate the objective function for each secretary bird
Identify the best solution (**BestBird**) based on fitness
for $t = 1$ to T **do**
    Update the best secretary bird position
    for $i = 1$ to N **do**
        Exploration:
        if $t < \frac{1}{3}T$ **then**
            Calculate new status of the i-th secretary bird using initial exploration strategy
        else if $\frac{1}{3}T \leq t < \frac{2}{3}T$ **then**
            Calculate new status of the i-th secretary bird using intermediate exploration strategy
        else
            Calculate new status of the i-th secretary bird using final exploration strategy
        end if
        Exploitation:
        Generate a random number r
        if $r < 0.5$ **then**
            Calculate new status using first exploitation strategy
        else
            Calculate new status using second exploitation strategy
        end if
    end for
    Save the best candidate solution found so far
end for
Return BestBird as the optimal solution

(**c**)
